# Inattentive Perception, Time, and the Incomprehensibility of Consciousness

**DOI:** 10.3389/fpsyg.2021.804652

**Published:** 2022-02-08

**Authors:** Jürgen Krüger

**Affiliations:** AG Hirnforschung, Freiburg, Germany

**Keywords:** inattentive perception, consciousness, energy, filling-in, identity, qualia, time

## Abstract

Cerebral energy supply is insufficient to support continuous neuronal processing of the plethora of time-constant objects that we are aware of. As a result, the brain is forced to limit processing resources to (the most relevant) cases of *change*. The neuronally generated world is thus temporally discontinuous. This parallels the fact that, in all relevant microscopic fundamental equations of nature, temporal *change* plays a dominant role. When a scientist calculates a “solution” to such an equation, integration over time is an essential step. The present Hypothesis expresses that the step from neuronal activity to phenomenal content of consciousness is reflective of a (phenomenal) “solution:” the main source of the incomprehensibility of consciousness is proposed to result from the introduction of phenomenal time-constant entities. These are “filled-in” *via* integration, even though neuronal data only exists for *changes* to these entities. In this way, a temporally continuous picture of the world phenomenally appears. Qualia are “initial conditions,” which are required for integration and cannot be deduced from *present* data. Phenomenal “identity” (vs. “high similarity”) is related to qualia. *Inattentive* visual perception, which is only rarely investigated, offers insights into these relationships. Introspectively, unattended vision appears rich because percepts are cumulated over long time spans, whereas attentive perception relies purely on *present* neuronal signals. The present Hypothesis is that a brief neuronal activity can *signify* long-lasting and constant phenomenal content of consciousness. Experimental support is presented that comes from discrepancies between neuronal activity and perception: transient neuronal responses to sustained stimuli, “filling-in,” change blindness, identity vs. close resemblance.

## Introduction

The present communication is concerned with Chalmers' “Hard Problem” of consciousness (1995, 2018): essentially, “why and how do physical processes in the brain give rise to conscious experience?” or, expressed otherwise: “an organism is conscious if there is something that means what it is like to be that organism and a mental state is conscious if there is something that means what it is like to be in that state.” To solve the hard problem, one must unite neuronal processing, which obeys the principles of natural science, with reported or subjectively felt content of consciousness. The difficulty is similar to deducing the concept of (monetary) value from an examination of the material properties of gold. Chalmers (1995) argues that the hard problem cannot be solved using the methods of cognitive science and neuroscience; in addition, there are numerous further problems related to consciousness that are described as “easy,” although they are extremely difficult to tackle in practise.

One of the main approaches to these “easy” types of problems is to determine a set of similar experimental paradigms, some of which can be reliably associated with consciousness and some of which cannot, and to draw inferences from the corresponding differences. Many findings, such as the relevant cerebral areas, brevity of initialisations of conscious events, ways of neuronal processing, computational principles and the importance of top-down signals from the prefontal cortex (Flohr, 2000; Dehaene and Naccache, 2001; Haynes and Rees, 2005; Tononi, 2012; Koch et al., 2016; Lamme, 2018; Brown et al., 2019; Mashour et al., 2020) do not conflict the present proposal.

While the hard problem will not be solved in the present communication, it will be related to other problems that merit a description as “hard” so that the overall number of independent hard problems is reduced. I consider the origin of the concept of “time” and the truth of mathematical relationships to be manifestations of such problems. Moreover, a further situation can qualify as that of the “hard” type, which is closely related to what McGinn (1991) refers to as “cognitive closure:” I call this the “back reference dilemma of consciousness.” This will be addressed in the section “Theoretical Considerations” (see also the section “Terminology and Definitions”).

Other difficulties become apparent when one realises that a wealth of time constant elements are perceived in our complex structured world. Nonetheless, not even a layperson would believe that all of this could be constantly signalled for hours on end by time constant neuronal activity. How is it possible, then, that phenomenal experience comprises a host of long-term, constant elements? This directs the focus of the present communication to the temporal domain, which is supported by unique insights taken from research on inattentive visual perception.

## The Hypothesis

Limited metabolic energy is a paramount problem for the brain: given the limitations in energy supply, the brain is unable to represent constant, long-lasting features of our physical world in the form of constant, long-lasting neuronal activity. In order to cope with this deficiency, neuronal activity is devoted to the processing of *change*.

This alone, however, does not solve the problem. Consequently, three further measures are taken: (i) Instants of change that require processing occur as rarely as possible: periods with weaker changes are treated as constant so that no processing is required and only significant changes prompt neuronal activity. (ii) Changes that can be traced back to the activity of the brain itself are disregarded. (iii) In complex scenes, only *components* of change are processed: the unchanged parts of a scene may remain untreated or, in other cases, the derivation of a higher concept is not neuronally executed, i.e., there is no corresponding change, while changes occur at a lower conceptual level (Kouider et al., 2010).

Nevertheless, time-constant features of the environment (e.g., for spatial orientation) need to be taken into account; procedural memory stores such features. When actions involving orientation are required, neuronal activity is guided by this type of memory. For reasons of limited energy resources, however, constant and continuous retrievals are not possible.

In this way, the neuronal representation of the world is sparse and temporally discontinuous.

The essential role of consciousness is to provide the phenomenal appearance of long-lasting, constant features that intervene between instances of change so that a continuous world can be subjectively experienced. There is no neuronal activity underlying constant periods. One should not dismiss such a relationship from the outset: For the phenomenal filling-in of constant periods, no new data are necessary: only the data deriving from neuronal activity at the beginning of a constant period is needed. I propose that phenomenal experience without underlying, concurrent neuronal support is possible since phenomenal content is not a physical entity.

The essence of the present Hypothesis is: brief neuronal activity can have long-lasting and constant significance.

## Terminology and Definitions

All our bodily organs, including the brain, function by activating *procedures* that have different tasks. When problems related to the brain are studied in accordance with the same general principles of natural science as other organs (i.e., one of Chalmers' “easy” problems), one can determine how a given observable function is realised. Generally, procedures in bodily organs, including the brain, react to relevant external situations upon which they start a learned or inherited activity. No consciousness is implied. In the same vein, one can find brain-mediated reactions to visual stimuli or even throat muscle commands (“speech”) as a response to such stimuli. However, one *cannot* find traces of the subjective phenomenon of perception (which in everyday language is termed “seeing”). The great majority of visible entities that are perceived every day cannot be related to any observable reaction to a stimulus and, most certainly, one would not find memory traces of these reactions.

My understanding of *consciousness* is accurately expressed by Chalmers' sentences given in the Introduction. More detailed attempts to define “consciousness” and to divide it into various sub-types can be found in many texts, such as Chalmers (2007).

I take “consciousness” to be the result of the attribution of “*meaning*” or “*significance*” to neuronal processes (Orpwood, 2017). Significance appears subjectively as *phenomenal content* on the *phenomenal level* of consciousness. “Significance” is not a signal: no material receptor exists for it. The attribution of significance is not a valid operation in natural science. This restates Chalmers's (1995) “hard problem” in other words. More detailed attempts to define “consciousness” and to divide it into various sub-types can be found in many texts, such as Chalmers (2007).

The medical differentiation between “consciousness” and “unconsciousness,” which hinges on objective observations, will not be taken into consideration.

On the other hand, all “knowledge” is situated on the phenomenal level, from our everyday understanding of the world around us to the entire scaffold of natural science. The entity, or system, that attributes significance is termed here “*observer*;” this is the entity that “knows” and is a phenomenal entity, i.e., it is *not* a physical person (accordingly, the personal pronoun “it” will be used). Mere physiological activity in the brain and other organs, as well as the conclusions drawn from the output of organs or the behaviour of creatures, are not evidence of these organs or creatures as having “knowledge.”

A scientist cannot work unconsciously. Rather, one must be conscious in order to study consciousness. This is essentially circular reasoning, also referred to as *back-reference dilemma*. For the case of “time,” the dilemma translates into “One must attribute temporal significance in order to study how one attributes temporal significance.” If scientific reasoning employs the concept of “time” as a self-evident premise, then no valid insights can be expected from attempts to clarify questions related to the concept of “time.”

The sensory part of consciousness is “*perception*,” i.e., perception is not a physiological process. *Percepts* are thus the significances of sensory neuronal processes. In the present communication mainly visual perception will be considered.

Perception may be *attentive* or *inattentive*. I consider any sensory event to be “perceived” if it appears on the phenomenal level, irrespective of the nature of the experienced content, or whether attention, awareness or any other additional factors are involved.

Note that other scientists adopt wider definitions of perception by extending the usage of the term to neurophysiological sensory processes, while others consider “unconscious perception” or attempt to split it up (e.g., Vetter et al., 2000; Kiefer et al., 2011; Chirimuuta, 2014; Salti et al., 2015; Berger and Mylopoulos, 2019; Pizlo and de Barros, 2021; see also Lamme, 2004 for further details).

The *sensorium* is the ensemble of sense organs (eyes, ears, skin, muscle spindles signalling muscle effort). Inner senses, e.g., from the gut, are disregarded.

Temporal “change” is considered to be an event occurring within an infinitesimally brief instant, while the duration of a neuronal *reaction* to that change can range from a few milliseconds to several minutes.

A *quale* (plural: *qualia*) is the experienced quality of a phenomenal content. An example is the experienced quality of “redness” when an appropriately coloured stimulus is offered.

## Supporting Evidence

### Processing Energy in the Brain

The limitations of neuronal processing in terms of energy have not been investigated in the context of the present topic. Nonetheless, previous findings support the present assertions: the brain requires large amounts of energy. It always runs at maximal power consumption. Even a brief interval above average energy levels in a limited cerebral region needs to be balanced out by reductions in metabolism elsewhere (Clarke and Sokoloff, 1999). Lennie (2003) writes: “Even with only a few percent of neurons concurrently active in any local region, the metabolic burden would be unsustainable over all of cortex.” A consequence of this is that, if attention is devoted to a visual discrimination task, processing capacity is lower for other unattended processes (Bruckmaier et al., 2020). Since inattentive visual processes constitute by far the largest part of total perceptual content (see below), energy considerations make it unlikely that inattentive perception is mediated by *current* neuronal activity.

### Transient Neuronal Responses vs. Constant Perception

The purpose of many neurophysiological studies on visual processes is to determine the foundations of human perception. Some obvious discrepancies have been known since the first physiological studies of single neurones: a bounded, homogeneously illuminated area offered as a visual stimulus (e.g., a white sheet of paper) is perceived as constantly illuminated from its edge to its inner regions. In contrast, most neurones that signal illumination display strong levels of activation when their receptive fields are directed toward the edge of such a stimulus (Baumgartner, 1961), whereas activation is much weaker or even absent for the inner portions. An analogue situation is true for the temporal domain: when a light is switched on and remains constantly illuminated thereafter, it is perceived as constantly illuminated from the moment it is switched on. Once again, illumination-signalling neurones respond strongly at the outset of such a stimulus but their activities markedly decrease or vanish only a fraction of a second later. One sees illustrations of such transient responses to sustained stimuli in many publications (e.g., Krüger, 1979; Livingstone and Hubel, 1984; McLelland et al., 2010).

The need to explain the spatial or temporal constancy of visual perception led to a search for “luxotonic” neurones (Marrocco, 1972; Kayama et al., 1979), i.e., for neurones whose firings signal constant long-term illumination of an area. This quest was not successful: Marrocco (1972) notes that “ambient luminance information […] is, to some extent, sacrificed […] in favour of information about transients.” At higher levels of processing, apparently erratic components of excitations were found to be superimposed on responses (Kayama et al., 1979) or no response to longer lasting stimuli was observable. In a functional Magnetic Resonance Imaging study, Haynes et al. (2004) observed decaying neuronal activity to constant visual stimuli. Due to the limited temporal resolution of this imaging technique, a pronounced initial transient could not be observed.

For the spatial case, this discrepancy can have a different cause if the foveal vision is taken into account: many receptive field sizes in monkey foveal striate cortex are <10 min of arc wide (Dow et al., 1981), a value that can certainly also be assumed to be valid for humans. During careful fixation, the gaze direction of primates undergoes microsaccades of up to 1° (for a review, see Martinez-Conde et al., 2009), disregarding other types of small drifts. Thus, stronger neuronal reactions to spatial borders may be due to stimulation *via* small gaze shifts over borders, rather than by being enhanced by the centre-surround antagonism of retinal receptive fields. For the sake of the present argument, however, the effect is the same.

In conclusion, the (usually perfect) constancy of perception of a physically constant spatial or temporal stimulus is not understood on the basis of neuronal activity.

### Spatial “Filling-In”

Although the focus here is on the temporal domain, an analogue case will first be considered in which the relevant phenomenon occurs in the spatial domain: It has long been known that a border on the retina between two differently illuminated regions is ignored when that border is absolutely fixed on the retina. This stands in contrast to careful (natural) gaze fixation, which involves tiny shifts of the image (Ditchburn and Fender, 1955; Martinez-Conde et al., 2009). Should one view, for instance, a large yellow spot on a black background under natural gaze fixation, one normally perceives that homogenous yellow spot on a background. However, if within that area a smaller green spot is shown and its borders to the yellow are absolutely fixed on the retina, perception of the green spot rapidly fades away after onset and, once again, only a completely homogeneous yellow spot is perceived (Krauskopf, 1963; Gerrits and Vendrik, 1970; Nerger et al., 1993; Komatsu, 2006). The reader can experience an approximate demonstration of this effect in a collection of optical illusions (Bach, 2002) in which “disappearance of a spot” is usually reported. However, even scientists often miss the crucial point which is that one perceives a physically non-existent background colour in place of the vanished spot. Monkey colour-selective neurones have been shown to continue to respond to green (more weakly than at the borders) in such cases (Von der Heydt et al., 2003). Given that the visual systems of monkeys and humans are comparable on the primary neuronal level, it can be concluded that the “fading” perceived by humans is not due to a lack of neuronal signals from the interior of the green area.

For purposes of perception, one can conclude that the visual system only uses border information, such as “left side black/right side yellow.” If no further border exists on the righthand side, “yellow” remains valid until another border is encountered, irrespective of the neuronal responses from receptive fields directed toward the inner parts of that area. Obviously, if the yellow/green border is ignored because it is stabilised on the retina, “yellow” remains valid even in the green field. Mathematically, this mode of operation (“filling-in”) corresponds to *integration* over the distribution of neuronally signalled spatial light *changes*.

The neuronal border descriptions, “left side black/right side yellow,” and, farther right, “left side yellow/right side black,” bring about the perception of an uninterrupted yellow field on a black background. Although this situation involves a stabilised border, it is valid for all cases of normal perception: a yellow field, viewed under normal conditions, is perceived as a completely filled yellow area. Although exposed to yellow light, the neurones with receptive fields in the middle region of the yellow field do not contribute to that perception; their reduced or absent firing is possibly recruited for behavioural purposes (O'Regan, 2016). For perception, the only fact that counts is the *absence* of any border within the field. The detection of absence is a neuronal affair: it may be crude and noisy but, whenever there is no change signal (instead of no percept at all), the outcome is a spatially constant percept.

### Temporal Analogue of “Filling-In”

It is not so far-fetched to assume that the same principle is equally valid on the time axis given the close similarity between neuronal reactions to spatial and temporal illumination changes, and the benefits to energy economy in both cases: suppose after darkness, a yellow light is switched on and remains constantly illuminated. In such a case, for the purposes of perception, the visual system only uses the “temporal border” information—“first black, then yellow”—that is available after a few dozen milliseconds and, if no further “temporal border” is detected, “yellow” remains valid until another change is encountered. Thus, in analogy to the spatial case, the perception is “black, then long-lasting constant yellow;” the subsequent reduced or absent firing has no effect on perception but may be needed for procedural seeing, i.e., for visually guided behaviour. An overview of this field of research can be found in Spillmann (2011a,b).

A “temporal border” should be the output signal of a general detector for temporal image changes of all kinds (luminance, wavelength and movement). A unified general detector for all of these purposes has not been described thus far but could be assembled from a few types of more specialised detectors (line inclination, movement direction, colour, spatial frequency, etc.; Hubel and Wiesel, 1977; Livingstone and Hubel, 1984; Tootell et al., 1988). Similar to the spatial case, this detector may be fairly inaccurate and noisy so that objectively quite dissimilar scenes appear to be identical on the phenomenal level.

The idea is that, first, neuronally executed temporal differentiation occurs, followed by phenomenally executed temporal re-integration (see the section “Integration over temporal change”). This yields an unchanging value as expressed by the Hypothesis.

### Effects of Sensorium Movements

A major problem that has been recognised since the advent of modern neuroscience is the ubiquitous experience that one perceives the *same* global scene when one repeatedly returns to the same environment or when there is a change in the direction of gaze. Inspired by studies on invertebrates and fish, respectively, Von Holst and Mittelstaedt (1950), Von Holst (1954; “reafference principle”) and Sperry (1950; “corollary discharge”) recognised that there must be a potent mechanism that keeps track of the position and movement of one's own sensorium. At that time, the continuous *neuronal* subtracting of these self-generated signals was proposed but this would considerably overload the processing capacity of the brain. The present proposal differs from these ideas insofar as only brief instants of change are of concern. When these changes are derived from self-generated neuronal activity, they are eliminated from the ensemble of changes that require analytical processing. More precisely, it is not that their neuronal *effects* are subtracted. Rather, their significance is disregarded as evoking a change *on the phenomenal level*.

Some secondary mechanisms may contribute to the same aim: for instance, one's entire body may move passively. In such cases, one can often rely on some tactile hints from which it is clear that it is not the environment that is moving but one's own body. Another example is that the substantial eye movement component of the eliminatory mechanism is recognised optically *via* typical, sudden shifts of the entire visual scene. A corresponding type of neuronal activity is the periphery (or shift) effect in the afferent visual system (Krüger and Fischer, 1973; Krüger, 1977).

### Change Blindness

“Change blindness” is a striking phenomenon (Blackmore et al., 1995; McConkie and Currie, 1996; Hayhoe et al., 1998; Pinto et al., 2017): one continues to perceive an *un*changed visual scene even when large modifications to that scene have occurred. The necessary condition is that these manipulations occur during a saccade, i.e., within its approximate duration of 60 ms. Otherwise, scenic manipulations are easily noted. Apparently, the performance of the mechanism that functions to eliminate the effects of movement in the sensorium is not very accurate: when bodily or eye movements occur, there is no further examination of the quality of the correspondence with sensory changes; only occurrence within the relevant time span is sufficient. Quite generously, the system delivers the message “no change in the outer world.” This effect can even be seen in certain types of optical manipulations (O'Regan et al., 1999).

The drawback is that there is a small temporal window in which the system can be duped: genuine changes that occur during the brief duration of a saccade inevitably register as “no-change” cases. Thus, a previously initiated percept can erroneously continue to persist despite a true change in the outer world. This is in accordance with the Hypothesis: a percept initiated by brief neuronal activity hours ago can continue to persist.

In an experiment linking peripheral colour desaturation to gaze movement (Cohen et al., 2020) most subjects did not note these desaturations; instead they felt that the entire scene was fully coloured all the time. The authors state that “these results show that during active, naturalistic viewing conditions, our intuitive sense of a rich, colourful visual world is largely incorrect.” Instead, I interpret the results in a similar way as the above change blindness linked to saccades. If one has a severely limited processing power, this is the best one can do, with the risk of error in cases of expected constancies turning out to be unfounded. The remaining big problem is the concern of the present communication: how are these long-lasting constancies treated? In Cohen et al.'s report it is not clear whether it is energetically more advantageous to generate correct or incorrect percepts, and where the percepts of physically inexistent items come from.

### Inattentive Perception

I perceive the world around me throughout the day whenever there is enough light, my eyes are open and I am awake. Moreover, the large majority of what fills my visual field is *identical* over long periods of time, e.g., the furniture at home, the buildings along my daily walk to work or a lecture hall while I listen to a presentation.

Perception is *attentive* during the brief, initial period in which there is accompanying neuronal activity. For (possibly very long) periods thereafter, neuronal activity fades. Perception then becomes *inattentive*. According to the Hypothesis it is not accompanied by any content-carrying neuronal activity.

A description of a prototypical, everyday scenario may be helpful: there is a large set of bookshelves that sit opposite my desk, which I inattentively perceive to be identical, stable and optically in-focus for many hours. I cannot switch off this percept except by closing my eyes or averting my gaze. I cannot inattentively select a visual target. Without this type of uninterrupted inattentive perception I would feel blind. If I am asked to pick out a particular book, I have to switch to attentive perception which I can limit to that book. The preceding state of inattentiveness is difficult to describe: there is no hole in place of the book in my visual field, yet inattentive perception cannot be used to find the book in question.

The description is reminiscent of the homunculus scenario in which a tiny humanoid located inside my head “watches” a projection of the outside world on an internal screen. This arrangement is usually considered a fallacy (Tibbetts, 1995) but it nonetheless demonstrates that the entire visual field is present at any one time. This corresponds to inattentive perception. The task of the homunculus is to select a target for closer examination or to initiate visually-guided action, i.e., the homunculus directs “attention” to a part of the whole image.

One can equate Block's (1995) “access consciousness” or “A-consciousness” with those brief intervals in which a change occurs. The neuronal system reacts to a change and an ensuing action is then elicited. I equate this with “attentive perception.” Inattentive perception thus, corresponds to Block's P-consciousness. Perception is regarded as “attentive” when there is concurrent neuronal activity and as “inattentive” if there is none. However, in contrast to Block's view, there are *not* different types of phenomenal content. I do not believe that attentive perception can have any causal effect. The apparent concurrence of neuronal activity and attentive perception results in the unfounded assumption that it is phenomenal content that plays a causal role. If one accepts my assertion that there is only one kind of perception, it follows that any causal effects result from neuronal activity alone.

It is important to note that “concurrence” in this discussion can admit some temporal blur (Hayhoe et al., 1998; Block, 2007b). However, this is not meant by the Hypothesis: there can be hours or days between the instant of an experienced (constant) content and the underlying brief neuronal activity.

Clearly, most of the visual impressions one has throughout the course of a day are left unattended. This may even be true of foveal vision, for instance, if I were to sit in an armchair with my eyes open and not attend to any particular object, while instead focusing my attention on a radio broadcast.

A limitation of inattentive perception is that the sensorium must be appropriately positioned, i.e., in principle, the stimulus must be within the visual field. This implies that at any instant, I *should* be able to attentively examine the stimulus in question. Lamme (2020) describes such cases as “potentially visible.”

All things considered, it is surprising how little research is devoted to the inattentive part of perception. A remark by Van Boxtel et al. (2010) elucidates the issue: “attention primarily reduces the complexity of incoming input so that the brain can process it online and in real time.” Note that this comment fails to mention that the full visual scene needs to be reduced, which the authors assume is not processed online (but which is nevertheless inattentively perceived).

Admittedly, systematic studies on inattentive perception are impeded by a lack of a trigger instant that allows for the establishment of links between measurable physiological variables and inattentive perception. In addition, subjects participating in scientific studies on inattentive perception must, as a minimum, pay attention to a display that conveys the experiment. Consequently, it is not surprising that reference to inattentive perception in the literature is rather indirect: Lamme (2004) notes that one can be aware of an event that is nevertheless left unattended; he designates such events unstable and vulnerable. He further categorises them as “unattended” and “potentially visible,” yet not reportable or accessed “right now” (Lamme, 2020). Otten et al. (2017) write, “Introspectively, vision appears rich and detailed for most of the visual field. How can this seemingly rich visual experience result from limited retinal output? Perhaps people's actual experience is rich and detailed because the brain supplements the details and richness when bottom-up input is poor.” Blackmore et al. (1995) consider the richness of inattentive perception to be an illusion, whereas Odegaard et al. (2018) invoke a strange, psychology-based mechanism of “inflation.” Bronfman et al. (2014) posit a possible bottleneck between “seeing” and “reportability” for the transition from inattentive to attentive perception. Block (2007a) points out that “a minimal neural basis is a necessary part of a neural sufficient condition for conscious experience.” Naccache and Dehaene (2007) observe that “we all have the illusion of seeing a world in full colour although colour-sensitive cones are absent in the periphery of our retina,” while Kouider et al. (2010) note, “When observing a complex visual scene, we feel that we have a rich visual experience even if we can report only a few elements.” Dehaene et al. (2006) consider a temporary process called “preconscious.” However, it does not share the characteristics of inattentive perception as given above. In sum, all of these observations consolidate the issue at hand: inattentive perception has an utterly incomprehensible component.

The description so far has been somewhat over-simplified. There is evidence that brief, current sensory signals first feed a centrally-generated Top-Down signal, from which perceptual content is then derived. Thus, it is more than just a copy of the current retinal image. Rather, some generalisations are applied (Landman and Sligte, 2007; Hatfield, 2014) so that, in the ensuing period of constant inattentive perception, the identity of the target can be experienced despite perspectival changes resulting from one's own movements. Indeed, attention is known to involve extensive feedback loops between higher-level areas in the brain and sensory cortices (Dehaene et al., 1998; Silvanto et al., 2009). The interleaved Top-Down mechanism also allows for the direction of attention to a target within a *constant* visual scene as if there were a true change to that scene.

There are various ways of confusing inattentive perception with other visual phenomena: what I do *not* mean is mere sensory neuronal processing without any accompanying phenomenal content. It is a remarkable peculiarity of vision that situations *devoid of* phenomenal content cannot occur during the day. In contrast, sensory neuronal processing without any accompanying phenomenal content frequently occurs in tactile perception: there is no continuous perception of the pressure between one's feet and the ground while standing, yet continuous tactile sensory signals are necessary for the maintenance of equilibrium of the body.

Similarly, what is not meant by inattentive perception is a situation in which a test subject reports “seeing nothing” in a limited field of view, while neurones in the brain of this person react to the stimulus or show some other physiological effects (e.g., Mei et al., 2015; Silverstein et al., 2015). In my terminology, this corresponds to *attentive perception of nothing* since the test subject was paying attention to a limited field in the display. In contrast, inattentive perception implies that the person is clearly “seeing something” but is not paying attention in a visual sense, e.g., while he or she is involved in a discussion.

Inattentive perception does not appear in a “plethora of phenomena and paradigms in experimental consciousness research” (Kiefer et al., 2011). Dehaene and Naccache (2001), for instance, maintain quite generally that attention is a prerequisite for consciousness.

However, what else could “inattentive perception” be other than a process belonging to consciousness given that it is both phenomenally experienced and not objectively observable? At a minimum, it is remarkable that an attentively perceived object appears to be *identical* to the same object when it is inattentively perceived.

### Disease

A possible exception of uninterrupted visual perception is the case of patients with cerebral lesions leaving only the dorsal processing stream in the cerebral cortex intact (the “Where”- or, rather, the “How”-System; Goodale and Milner, 1992; Ungerleider and Haxby, 1994; Milner and Goodale, 2008). These patients can use vision for the guidance of behavioural acts but they are unable to report on what they see. This is interpreted as the absence of perception.

A long-standing view (Frith, 1979) regards schizophrenia as a disease of consciousness. According to the present view, disturbances in the attribution of time would be expected. In schizophrenics, the concept of “time” may indeed decay (Fuchs, 2007; Giersch et al., 2016; Martin et al., 2018) although Giersch and Mishara (2017) also found disturbances in non-conscious contexts.

## Theoretical Considerations

According to the Hypothesis, the neuronally-represented world is temporally discontinuous, whereby the felt experience of temporal continuity is provided by consciousness.

It may be helpful to provide an intuitive impression of the Hypothesis. Take a map of an ocean as an example in which water depth is represented by shaded contours and each shade represents a range of depths. Of course, in reality, these depth values are continuous. A similar (one-dimensional temporal) picture holds for the visualisation of the Hypothesis. Continuous variations of a given parameter (for instance luminance) at a given place phenomenally appear as a series of steps with sudden ascents and descents, with extended “flat” sections in between. One could imagine such a visualisation for many spatially neighbouring places. The resulting spatiotemporal landscape (“contour line representation”) grossly resembles the original scene: the more pronounced hills and valleys appear approximately at the correct places and times and smaller variations are erased. This is the phenomenal image. In contrast, the neuronal image is much poorer: there are only non-zero values at the step ascents or descents, and zero everywhere else. The non-zero values quantitatively indicate the magnitudes of the steps (a similar mechanism for the spatial domain is disregarded here).

If these images were transformed into pixelated representations, one would believe that there is much more information in the phenomenal image than in the neuronal image. In reality, no more than a computational rule is added to the neuronal image in order to reach the phenomenal one: “Apply the three additional measures contained in the Hypothesis, and then form a temporal integral over the neuronal image.” Of more importance is the principal reason for the richness of inattentive perception: the effects of neuronal signals are limited to instants of occurrence, and they cease when activity ceases. In contrast, inattentive percepts stem from a range of past times and, in cases of constancy, once they are generated they do not disappear unless the next change occurs. They therefore encompass pictorial details that have been neuronally collected throughout prolonged time spans in the past. This makes them much richer than the attentive percepts that depend only on *current* neuronal activity.

A problem is the scientific status of the above computational rule: the integral can by no means be executed neuronally since it would lead back to the energetic overload that just had been avoided. Instead, one can say: “With the appearance of a change in the neuronal image, automatically the temporal integral until the next change is fully defined.” With each change, the integral is specified as a kind of general truth similar to that of 3 + 4 equals 7 which is considered to be true even before someone calculates it.

The admissibility of performing mathematical operations, including integrations, is not a part of natural science proper. I suggest that the status of “mathematical truth” should be associated with “qualia” rather than with cerebral processing principles. However, the topic is not pursued here.

An additional issue is that the above computational rule is not applicable to *any* neuronal activity. There must be some constructive or organisational element (which, in principle, should be scientifically detectable) that determines *which* excitations a temporal integral must be associated with. The approach by Tononi (2012) and Tononi et al. (2016) could be related to that question. In any case, the prefrontal cortex should play a major role (Mashour et al., 2020). Also it is no trivial statement that apparently no other organ than a brain can contain such an element.

It may now be useful to have a look on the foundations of natural science.

### Natural Science

All fundamental laws of nature (named after Newton, Maxwell, and Schrödinger) are differential equations in time, i.e., there is an emphasis on temporal change. The equations are also relevant for biological and neuronal processes.

It may be helpful to illustrate the significance of “temporal change” in more familiar terms since it only regards *immediate* successions: Instead of saying, “the rugby ball went through the goal posts because it was kicked in the right direction,” the laws state “it passed between the posts because it was already nearly at that target area a tiny (infinitesimal) instant before, and at that instant it moved in the right direction.” The point is, at that later instant in time, the kicking event no longer exists and hence cannot have any effect. Whatever elements of the past that are necessary for lawful temporal evolution have to be carried along step by step. In this way, the values of the physical variables necessary for lawful temporal progression are available at any given instant and this instant contains all the details required for the next step. It is implied that there are no relationships that bridge extended time spans, irrespective of what happens in between.

### Neuronal Processing Without Consciousness

What is valid for physics is also valid for the working of any organ, namely to operate in the present. It is also true for a “purely physiological” creature, i.e., a creature that has no consciousness (the statement does not mean that an animal cannot have consciousness. It simply means that I limit such considerations to physiological processes. For more on the topic, see Mallatt and Feinberg, 2021). Certainly, such an animal can *learn* to cope with problems but it neither *knows* that it learns nor that it was unable to solve a given task before having learnt it. Whatever it has learnt will be executed as if it had always dealt with the problem in this “new” way. Similarly, the creature may have evolved a neuronal procedure to “predict” an upcoming danger. When such a procedure is set in motion because of appropriate indicators in the environment, the animal does not know that the effect will be the avoidance of danger. Afterwards, when the creature has succeeded in avoiding the hazard, it does not recognise that this is a direct consequence of it earlier initiating the correct procedure. So far, this description corresponds to Hoerl and McCormack's (2019) “temporal updating system.” The animal cannot grasp what “repetition” is and therefore cannot carry out investigations in the human sense since this would depend on comparing present results to those obtained under similar conditions in the past. Nonetheless, the animal's performance will improve following repeated (and successful) behaviour. In summary, such an animal is fit for life and survival and its behaviour demonstrates intelligence, even though it lacks phenomenal knowledge.

Thus, to only be concerned with “change” would seem to be sufficient, whereby “change” means some kind of neuronally detectable interaction. Why be bothered by the “something” that fills the intervals between “changes”? What is this stuff that supposedly exists between changes?

### Integration Over Temporal Change

Consider a scientist who wants to apply the fundamental equations of nature to a concrete problem. While the equations provide an insight into nature's ultimate principles, it is difficult to intuitively grasp how they apply to individual cases. A major task of the scientist, therefore, is to introduce individual circumstances and then “solve” the relevant equation. Here, only a simple case, the switching on of a light, will be considered. It is not necessary to expose the fundamental equations responsible for these cases. The only necessary fact is a temporal change: in the case of luminance, that change has a defined magnitude of, let's say, 304 units.

In the present case, the scientist must execute an integration over a range of interest that extends from before the switching on of the light until some instant well after, when the light is constantly on. The change values remain at zero except for that single instant at which the light is switched on. The result of the integration is a step function along the time axis, which is at zero before the switching on and at a constant level of 304 units thereafter.

The scientist's “solution” reproduces the phenomenal content of consciousness experienced by a normal person, including that of a prolonged time course, yet the calculation rests only on a single “change” value measured at a particular instant.

Usually, the scientist does such an analysis in non-real time by relying on stored data but, in principle, it can also be done in real time without affecting the results. At any given instant, he/she needs only the current value of the integral computed and the value of the change that needs to be added to the integral. Although it is clear that an integral over time always depends on the past in a cumulative way, for the special case of long-lasting constancies with occasional brief changes, it is worth emphasising two points: firstly, an integral is a (partial) memory of the last change. Secondly, as already stated above, no computation is required during periods of constancy and no new data are needed. The results are “known” immediately after the last change and they remain valid until the next change occurs.

This description comes very close to that of consciousness: at the instant of an intensity change from zero to 304 units, visual neurones react briefly and the percept “light is on at 304 units” begins. At that instant, perception is attentive, which means that there are neuronal excitations related to the light change that can be used for further processing. At all subsequent instants, there is no change. Therefore, following the next infinitesimal step in time, the percept remains at 304 and is now inattentive. This continues until the neurones signal another change.

I return to the Hypothesis that says that a brief neuronal process can give rise to a prolonged constant percept. However, at any instant there is no prolonged time span in which future percepts could be located. Rather, the “observer” experiences that percept successively as time progresses. The integration machinery (whatever that is) needs no direct reference to the last non-zero change. In cases of long chains of zero change, each integration hinges only on the previous zero change. All of this is phenomenal and no neuronal processes are involved.

Another peculiarity of differential equations of all kinds has so far been pushed aside: if the magnitude of a variable changes from zero to 304 units, the effect is the same as if the variable changed from 100 to 404 units. Thus, a problem remains: since only the magnitude of *change* is relevant, absolute values are missing. On the other hand, it is the absolute values that one experiences, i.e., one experiences luminance, and not “the difference of luminance to some previous value.” This leads to “initial conditions” and “qualia.”

### Qualia and Identity

Every scientist familiar with solving differential equations knows that he/she must provide initial conditions; they cannot be deduced. A result obtained at any given instant has no absolute value but is relative to a preceding result, which is located an infinitesimally small time-step before the instant in question. Obviously, it is impossible to go back to times immemorial in order to find the very first *absolute* value of a variable.

The proposition here is that the quality of an experience (“quale;” plural “qualia”) *is* an initial condition. Qualia are inaccessible for the same reason as initial conditions are inaccessible; they must be provided by the scientist when integrations over “change” are applied. The integral derives the actual experience of a red light partly from a past instant when the light was switched on, and partly from the time of the ontogenese of the brain when the quality of the feeling of “redness” was established. All this implies that it makes no sense to attempt to explain qualia on the basis of *current* data.

Thus, the way in which “luminance” or “red” is experienced depends, in some respects, on an unknown, remote past. On the basis of the fundamental equations, one would have to conclude that, in nature, the only variable is “change in redness” or the appearance of “red” in a scene where no “red” was present before, whereas its temporal integral, namely “red,” exists only for purposes of human understanding.

In fact, initial conditions dominate phenomenal content of all kinds in a much more general way. For each component of a complex scene, they are fixed at the associated last change. Not all of the perceived properties of my screwdriver are determined by neuronal analysis each time I take it out of my toolbox. Some of them may have been fixed during a brief retrieval from an earlier episodic memory. Later, when I use that tool, only its displacements and movements are signalled as “changes.” The initial conditions for the diverse components of the percept of the screwdriver secure its constant identity over time.

In the same way, what an electron is depends on some initial conditions in an unknown remote past; one cannot determine by *current* measurements why it has these and not other characteristic properties. Also its identity over time, qualifying it as a “particle,” stems from a phenomenal integration. It is a reference to the last change even if it occurred at times immemorial.

An important point is that the relevant change is a *single* event: identity arises from temporally separated phenomenal references to a *single* neuronal process, namely the initial condition. In contrast, purely neurophysiological sequential processes can only yield impressions of close resemblance: recurrent cortical processes (see Lamme, 2006), repeated retrievals from memory, classifications (Catenacci Volpi et al., 2014), similarity above a certain threshold (Decock, 2018) or references to attractor networks (Mozer, 2009; Catenacci Volpi et al., 2014; Orpwood, 2017) cannot explain “identity.”

One should realise that repeated behavioural actions are never identical and the same is also true for *neuronal* activity that accompanies repeated situations. If one eats soup with a spoon, it makes no sense to aim for perfectly identical movements or for perfectly identical neuronal representations of the spoon. The idea that this spoon *now* is identical to the same spoon half an hour ago is highly artificial. As long as the purpose of each repetition is fulfilled, there is no incentive to aim for movements of a highly similar or identical nature. For an overview of that matter, see Debner and Jacoby (1994). Thus, in nature, there is no “identity.” There is no need for exact replication, neither in terms of behavioural action nor neuronal activity.

This lies in strong contrast to the phenomenal level of consciousness: the remarkable feature is that brief signals can generate significances that are experienced as *identical* percepts for long time spans. As such, they appear phenomenally in the outer world.

The experience of “identity” may be influenced by undesired effects: in some visual psychophysical studies, simple geometrical shapes are presented repeatedly on a computer screen. My personal feeling is that the attribution of “identity” is less convincing in such an experimental paradigm than under natural circumstances. I suppose that the potent natural mechanism “shadows should not be taken as real objects” extends its effects also to screen displays. One should not overlook that the task of the visual system comprises also to recognise that a solid object “the screen” is present which one easily recognises as identical over time.

### The Back Reference Dilemma

When any scientific experiment is done, the neurones in the brain of the scientist react to the findings. Understanding the results implies that he/she is conscious of the findings, i.e., they are subject to the transformation of neuronal processes into phenomenal content of consciousness. Consequently, scientists are victims of the “hard problem,” i.e., the results are subjectively understood but there is no appreciation of how this understanding comes about. This is systematically the case for scientific results as well as mundane reasonings of any kind; the entire scaffold of natural science is built upon this unsteady ground.

With this in mind, any attempt to study the hard problem of consciousness using the rigorous methods of natural science amounts to the investigation of a mechanism with the aid of precisely that same mechanism. Thus, even scientists who do not feel impeded by considerations about consciousness should at least acknowledge the restrictions imposed by consciousness on their scientific work. This is particularly important for the role of speech as a remedy for communicating with other individuals located in the outer world. At present, it is unsatisfactory that a person (including myself when writing this text) assumes that a speaker, listener, writer or reader (other than myself) can attribute phenomenal significance to the physical air waves of spoken language or the characters of a written text. Awkwardly, this assumption implies that the attribution of significance can occur in the outer world (in this case, another individual). It is doubtful that the scientifically approved biological similarity of all humans could act as a basis for this. On the other hand, it is certain that *significances* of neuronal processes cannot be transmitted between individuals. Therefore, one has to find out precisely what types of elements are transmitted *via* speech so that I (and no one else) can understand what I or others say, and on which neuronal processes my phenomenal belief that someone else understands what I say or write depend. One should be sceptical of the simple idea that speech largely reflects what happens in consciousness.

### Time

The central issue regarding the attribution of significance becomes apparent when the following simple question is asked: “How can a retrieval from episodic neuronal memory, as a *present* neuronal event, *signify* a past event?” or, more precisely, “…signify the temporal feature, ‘the past”'? In my opinion, such a question lies at the root of consciousness much more than the complexity problem discussed by others (see below). This is because, for the case of “time,” significance attribution cannot be circumvented: the normal work of the scientist becomes impossible if he/she rejects the attribution of “past” significance to the retrieved content of a biological or a technical memory. Nevertheless, quite generally, significance attribution is not a valid relationship in the realm of natural science.

However, when the task is to elucidate the status of consciousness that attribution would contaminate the investigations. A loss of “scientific comfort” is the consequence; the scientist's work becomes more wearisome: The concept of “time” can no longer be applied in equal measure to neuronal and to phenomenal events, and one cannot invoke causality, as it is usually done when intracerebral processes are studied.

That is not all. When one has internalised the idea that only the “present” exists as a single instant but neither the past nor future exist, one feels uneasy when encountering the statement that the “present” in the laws of nature comprise a temporal derivative, i.e., two infinitesimally separated instants are involved. These views can be reconciled as follows: take, for instance, an equation in which the position of a moving object plays a role. There could have been a forerunner to the presently used equation in which there was a compact variable “temporal change of position” (i.e., velocity), which is made up of both “position” and “change along a by-then unknown time axis” (see also Hinterberger, 2002). This compound is a variable whose magnitude can be measured by a tachometer. Such a measurement is done at a given instant; there is no indication of other such instants. In contrast, the introduction of “change,” i.e., of a temporal derivative, implies an infinitesimally small time step to a later instant. I propose that this is the origin of the “progression of time,” as well as the origin of “time.” Considered in reverse, it is conceivable that the step to an infinitesimally later instant only exists on the basis of a posteriori created concept of “time.”

For the present context, the fact is important that the main source of the incomprehensibility of consciousness is assumed to result from the introduction of time-constant entities, although neuronal data exist only for *changes* to those entities. All of this occurs on the phenomenal level but this level also serves as our view of the world. In periods without change, such physical entities are taken to exist only on the basis of extrapolation from a previous state but there is a lack of supporting data.

There is a further problem: the above description of integration in real-time lacks a means of phenomenally knowing how long ago the last non-zero change occurred. If one accepts the role of temporal integration as presented here, with its long periods of constancies, then a possible solution could be offered by a neuronal signal elicited by an event, such as the sunrise or “start of day.” Only one neurotechnical measure needs to be taken: a counter signal “sunset” or “end of day” must be suppressed. A sequence of neuronal “sunrise” signals would then lead to a stepwise increasing integral that can serve as a phenomenal time signal. Different types of repeating events nested in one another can be used in parallel. Note that such time signals are not available in *neuronal* terms, i.e., one cannot observe “time.” Nevertheless, they enable us to *know* how much time has elapsed since some arbitrary start event, with only the latter being a *neuronal* event.

Consider the statement “Five days ago I moved to a new domicile:” there are two components. The content “move-to-new-domicile” stems from some *neuronal* episodic memory. The process of its retrieval is a *present* neuronal event. One can assume that it has a neuronal attachment that impedes to confuse it with true reality, with the effect that phenomenally it appears as “not a real event,” together with the retrieved event itself. A coupling to the other component, namely those 5 days (“mental time travel;” Tulving, 1984; Suddendorf, 2013) might come about *via* some association of the above “arbitrary start event” of the integration. However, this will not be elaborated in the present framework.

## Discussion

Certainly the idea of a percept without neuronal support will provoke objections, all the more so since there is a strong intuitive feeling that perception (as defined in “Terminology and Definitions”) behaves like a physical signal, and that “to perceive” is the main purpose of the visual system rather than the guidance of behavioural acts. From these feelings arise assumptions that contradict the present proposals: Tyler (2020), for instance, assumes that “the neural substrate for conscious processing […] must have a spatiotemporal isomorphism with the experiential properties of consciousness.” McLelland et al. (2010) state: “it is generally hypothesised that driven responses underlie the perception of actual visual stimuli” (“driven” in this context means due to actual optical stimulation, as opposed to afterimages). When perception over extended time spans is taken into consideration, a succession of neuronal activity is assumed to occur in parallel (Melloni, 2015). Arguments based on “memory” could be put forth but only episodic memory would be applicable, if at all. A non-retrieved storage of any kind of memory is neuronally inefficacious; one would have to retrieve its content. However, a constant, never-ending readout of episodic memories would be energetically prohibitive.

Different views on incomprehensibility of consciousness can be found in the literature: If it is assumed from the outset that phenomenal content is concurrent with neuronal activity, for instance, when consciousness is held to require attention (see Mashour et al., 2020, for a review), or otherwise, then consciousness as such is no more than a particular way of neuronal processing. Yet, the enigmatic nature of consciousness is admitted by most authors but their views offer no hints as to which feature the incomprehensibility could be anchored.

The intellectual loans from the intuitively incomprehensible components of quantum mechanics will not be discussed here.

Thermodynamics is a branch of science in which reliable statements can be made (e.g., about gas pressure) even though not every microscopic detail is known (note that thermodynamic laws depend on what one knows, which is a phenomenal affair). In this vein, an avenue of interest to the problem of consciousness is offered by Friston et al. (2006) and Friston (2010). There is systematic, partial ignorance on the macro-level that can allow for considerations not existent on the microscopic level.

More specifically, Friston and co-workers propose a “delimiting” of the system by some boundary traced in the universe so that the system resists (at least for a while) the trend toward ever-increasing entropy (“heat death”). Heat death, in the long run, is the thermodynamic fate of everything. The system would have to carry out, at least approximately, the function of Maxwell's demon (Bennett, 1987), i.e., to guide all the individual molecules in just the right way so that the outcome is the minimisation of thermodynamic free energy. In order to successfully counteract decay, the system requires a model of the environment by necessity or, rather, must *be* such a model. So far, a closer connexion to the present views cannot be established but, nevertheless, the conceptual similarity between “to be a model” and “to signify” should be noted.

A psychology-oriented approach (Fuchs, 2020) invokes a type of back-reference dilemma (“circular causality”): a living entity is made up of the condition of its parts but this, in turn, is realised by those parts. The main problem with consciousness thus arises from a fundamental ignorance with regard to how known molecular interactions can organise the statistically extremely unlikely families of huge molecular ensembles that make up a living being. This type of organisational capacity is thought to be impossible *via* deduction from the laws of natural science. Effects in the physical world are then held to be caused by “embodied subjectivity.” Again, special cases of partial ignorance may play a role.

A number of other approaches suggest different roles for activity on neuronal micro- and macro-levels or, at least implicitly, an inextricable complexity (Tononi, 2012; Tononi et al., 2016; Fuchs, 2020; Rolls, 2021). One common aspect in many proposals, including those that invoke thermodynamics, is not explicitly stated: the source of incomprehensibility could be overcome if there were an immense analytical tool that would allow us to observe the dynamics and interactions of every molecule, every synapse and every neurone in the brain. However, even this would not resolve the enigmatic nature of consciousness; rather, new problems would be introduced, such as the loss of the concept of a macroscopic object to which one can attribute “identity.”

In summary, if one insists that phenomenal content behaves, with respect to time, in exactly the same way as physical entities, one only represses the question of the incomprehensibility of consciousness. More generally, it is deplorable that so little consideration has been given to the inherent limitations of natural science itself. Moreover, it is surprising that the back-reference dilemma is not seen as playing a major role as an obstacle to our understanding of consciousness.

### A Timeless World

The consideration of limitations to cerebral energy, which led to the Hypothesis, were done in terms of classical scientific reasoning. This encompasses Kant's (1787/1996) proposition to consider “time” as an a priori form of sensibility, i.e., there are no doubts about its traditional role. However, if the brain only has information about temporal change, how can knowledge about constant features exist? With this question in mind, one is less surprised by the fact that temporal change plays a dominant role in all relevant microscopic laws of nature. The constant features between changes appear only *via* the manoeuvre of “integration” whose validity is not covered by natural science.

In contrast, if one views the world from a neuronal perspective, then all constant portions are non-existent. The stimulus property “change” has only an infinitesimally short duration irrespective of the duration of the neuronal response. Therefore, a succession of changes is lumped together in an infinitesimally short interval. It is difficult to imagine a world without temporal extension. Nonetheless, in such a world the rules must be hidden that define the necessary step toward our familiar world in which constant sections are expanded. These expansions must fit together throughout the world so that they can be governed by a *single* time. In that world, an observer is a kind of “cursor” that indicates which tiny part of this huge timeless world is accessible. The fact that this depends on “change,” i.e., on (what then becomes) a temporal derivative, implies that there is also a rule in which “direction” it wanders together with the observed event through that expanded world.

The neuronal perspective casts doubt on the idea that “time” is anchored in natural science. Instead, in a hidden underlying “reality,” the world can be timeless. It is unsatisfactory that the observer determines, *via* “the present,” which parts of the world can be observed or, ultimately, which parts *exist*. This in turn implies that there are non-*existent* parts of the world.

“Time” establishes a link that connects parts of the world. It is not a physical interaction in the usual sense but gives an impression of the flavour of how phenomenal events are related to neuronal ones. It is also reminiscent of an essential aspect of the Global Workspace Theory (Baars, 1989; Dehaene and Naccache, 2001; Mashour et al., 2020): the workspace unites individual aspects of the world.

The idea of a timeless world is not so far fetched: several physics-oriented attempts are directed toward such issues (see, for instance, Kiefer, 1997: Does time exist?; Barbour, 1999, 2009: Time is superfluous; Briggs, 2015: How time emerges from timelessness; Mahler and Ellis, 2009: Current observation can generate facts even in the distant past).

Under these circumstances, it becomes highly doubtful that natural science can offer a sound basis for refuting the causal power of phenomenal content or freedom of Will (Kornhuber and Deecke, 1965; Libet et al., 1983; Soon et al., 2008; Schmidt et al., 2016). Rather, challenging the familiar aspects of “time” entails doubts about such “causality” and “freedom.”

## Conclusion

Inattentive perception is a strange phenomenon; no one can prove that I have it, I cannot react to it and it is difficult to describe. Fortunately, it provides a means of accounting for the incomprehensibility of consciousness, as expressed by the Hypothesis: normal scientific reasoning does not allow for relationships from cause to effect that bridge half an hour, irrespective of what happens in between. One can assume that the conclusions made with respect to “time” are also valid for the broader domains of consciousness: one can understand that our human view of the world, from electrons and celestial bodies to even flames, is dominated by the concept of the time constant “object.”

The proposals presented here hinge on a significant shortage of metabolic energy in the brain. There is just enough energy for the neurones to signal the most relevant cases of “change.” Constant features cannot be treated since they lack of relevance. For neuroscientists, it may come as a surprise that a similar deficiency is expressed by the laws of nature, in which the “change” of time-dependent variables play a dominant role and constant values between changes are only *deduced*. Perhaps this is what Chalmers (2018) has in mind when he says that the intrinsic nature of the physical may have a close tie to consciousness.

The step from neuronal activity as expressing *change* to the experienced normal world is essentially an integration over time. Additional support for this conjecture is provided by three facts: (i) integration requires initial conditions. These are scientifically inaccessible (the scientist just has to furnish them) and, for that reason, they are equated with “qualia.” It follows that qualia cannot be understood on the basis of *current* data. (ii) There is a dependency, for each variable, on only one initial condition for the whole period covered by the integration. Therefore, the part of a phenomenal content that is defined by the initial conditions remains *identical* throughout that period. (iii) Inattentive percepts are cumulated over longer time spans; they are thus much richer than attentive percepts.

While Chalmers' “hard problem” still awaits a solution, the present proposal sheds at least some light on the origins of the incomprehensibility of consciousness. The dominance of the role of “time” pushes that problem into the realm of the foundations of natural science. Neuroscience and psychology (Manzotti and Moderato, 2010) alone will not suffice.

## Author Contributions

The author confirms being the sole contributor of this work and has approved it for publication.

## Conflict of Interest

The author declares that the research was conducted in the absence of any commercial or financial relationships that could be construed as a potential conflict of interest.

## Publisher's Note

All claims expressed in this article are solely those of the authors and do not necessarily represent those of their affiliated organizations, or those of the publisher, the editors and the reviewers. Any product that may be evaluated in this article, or claim that may be made by its manufacturer, is not guaranteed or endorsed by the publisher.
